# Vaccine Policy in India

**DOI:** 10.1371/journal.pmed.0020127

**Published:** 2005-05-31

**Authors:** Yennapu Madhavi

## Abstract

India enjoyed early initial successes in vaccine development and indigenous production of vaccines in the public sector. But the country now faces a growing gap between the demand for and supply of essential vaccines.

Vaccines are important preventive medicines for primary health care, and are a critical component of a nation's health security. Although international agencies such as the World Health Organization (WHO) and the United Nations Children's Fund (UNICEF) promote global immunisation drives and policies, the success of an immunisation programme in any country depends more upon local realities and national policies (Box 1). This is particularly true for a huge and diverse developing country such as India, with its population of more than 1 billion people, and 25 million new births every year.

**Box 1. Local Realities and National Policies That Affect the Success of a Country's Immunisation Program**

- disease surveillance
- pathogen variations
- incidence levels that qualify for mass vaccination
- development and/or procurement of vaccines
- choice of technologies
- choice of selective vs. universal vaccination (even among childhood vaccines)
- logistics, cost-benefit analyses, and resource mobilisation

The current Indian market for vaccines is estimated to be about US$260 million [1]. India is among the major buyers and makers of vaccines, locally as well as globally, and has traditionally aimed at self-reliance in vaccine technologies and production. This article explores the trajectory of vaccine policy in India through its historical roots and institutional development, the gaps in demand and supply, the changing nature of the vaccine industry, and the emerging challenges in meeting national immunisation targets. [Fig pmed-0020127-g001]

**Figure pmed-0020127-g001:**
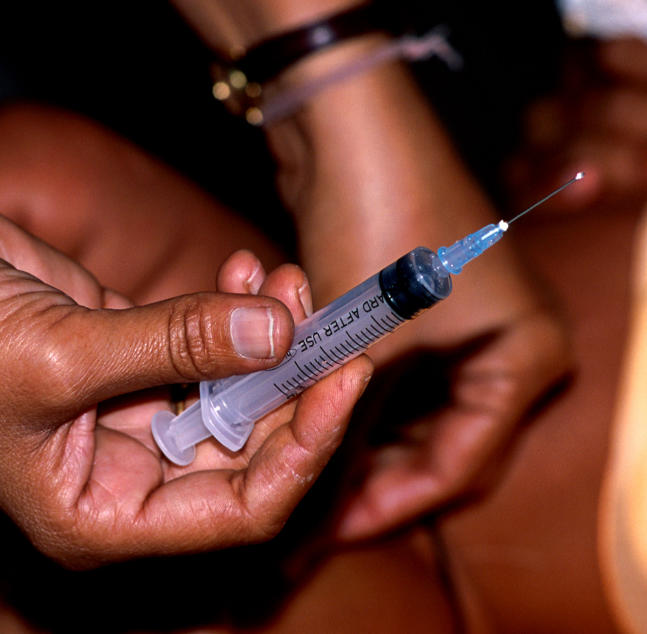
A nurse at Malipur Maternity Home (Delhi, India) prepares to vaccinate a child (Photo: the WHO/P. Virot)

## Early Origins

The history of vaccine research and production in India is almost as old as the history of vaccines themselves. During the latter half of the 19th century, when institutions for vaccine development and production were taking root in the Western world [2], the British rulers in India, concerned by the large number of their personnel dying from tropical diseases, promoted research on these diseases and established about fifteen vaccine institutes beginning in the 1890s. Prior to the establishment of these institutions, there were no dedicated organisations for medical research in India.

Haffkine's development of the world's first plague vaccine in 1897 (which he developed at the Plague Laboratory (Mumbai, India), later named the Haffkine Institute) and Manson's development of an indigenous cholera vaccine at Kolkata during the same period bear testimony to the benefits of the early institutionalisation of vaccine research and development in India [3]. Soon, Indian vaccine institutes were also producing tetanus toxoid (TT), diphtheria toxoid (DT), and diphtheria, pertussis, and tetanus toxoid (DPT).

However, the benefits of this early institutionalisation did not last long. The policies of the colonial government ensured that Indian scientists were not a significant part of this intellectual legacy. By the time Indians inherited the leadership of the above institutions in the early 20th century, research and technological innovation were sidelined as demands for routine vaccine production took priority [3]. By the time India gained independence in 1947, the Indian vaccine research and development (R&D) institutions were no longer on a par with vaccine technology development centres elsewhere. This is reflected in the fact that improved techniques for bacterial vaccines were introduced in India almost a decade after their introduction elsewhere in the world (Table 1) [4].

**Table 1:**
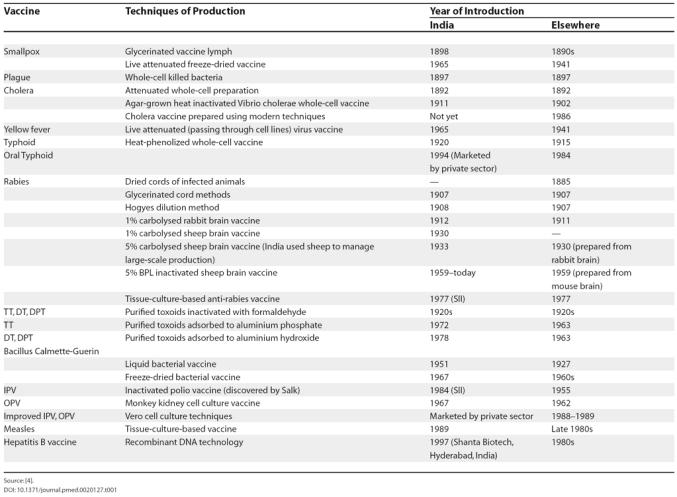
The Introduction of Vaccine Technologies in India and Elsewhere in the World

Source: [4].

What were the factors that led to the stagnation in vaccine development efforts between the time of Haffkine's success and India's independence? These included the pressures of routine production and service functions, financial constraints, lack of institutional mechanisms to foster and link up research and technology development, and the absence of an interdisciplinary approach. All these factors posed a threat to India's vaccine development efforts [5].

## Vaccine Policy in Independent India

One year after its independence in 1947, India became a member country of the WHO and eagerly aligned itself to the policies of the WHO and UNICEF. Many new Indian institutions were established with partial support from international organisations during the period 1950–1970.

However, after independence, it took three decades for India to articulate its first official policy for childhood vaccination, a policy that was in alignment with the WHO's policy of “Health for All by 2000” (famously announced in 1978 at Alma Atta, Kazakhstan). The WHO's policy recommended universal immunisation of all children to reduce child mortality under its Expanded Programme of Immunization (EPI). In line with Health for All by 2000, in 1978 India introduced six childhood vaccines (Bacillus Calmette-Guerin, TT, DPT, DT, polio, and typhoid) in its EPI. Measles vaccine was added much later, in 1985, when the Indian government launched the Universal Immunization Programme (UIP) and a mission to achieve immunisation coverage of all children and pregnant women by the1990s.

## Gaps in Vaccine Technology and Production: The Declining Role of the Public Sector

Vaccine requirements for India's EPI have been met mainly through the public-sector vaccine institutions, as was the case in most parts of the world until the 1980s. However, the Indian public sector failed to introduce new technologies of production (such as production of TT, DT, or DTP) or to expand production to become self-reliant in producing oral polio vaccine (OPV) or the measles vaccine [6]. Thus, even though successive governments have adopted self-reliance in vaccine technology and self-sufficiency in vaccine production as policy objectives in theory, the growing gap between demand and supply meant that in practice, India had increasingly to resort to imports.

In some cases, indigenously manufactured vaccines were stopped in favour of imported vaccines. For example, the Pasteur Institute of India in Coonoor indigenously produced polio vaccine during the period 1967–1977 with the help of seed virus from Dr. A. B. Sabin (who developed OPV) and with the approval of the WHO. However, the Indian government discouraged its production in 1977, alleging that one of the batches was virulent, and since then OPV has become one of India's major imports [4]. Subsequently, the Haffkine Institute was able to produce OPV indigenously, but this was mysteriously discontinued [5].

In 1987, the Union (federal) government's Department of Biotechnology established a new public sector unit, Bharat Immunologicals and Biologicals Corporation Ltd. (Bulandshar, India) with technology transferred from the Institute of Poliomyelitis and Viral Encephalitis (Moscow, Russia). The first phase of production was based on repackaging OPV imported in bulk from Russia. The aim was for OPV production to be completely indigenised in the second phase, within five years. However, the first phase continued (with imports) until the year 2000, when such importation supplied 70 million doses of OPV to UNICEF and earned a net profit of Rs 8 million. Yet, in 2000, the government declared Bharat Immunologicals and Biologicals a sick unit (a loss-making unit that is financially unviable), and its revival remains uncertain.

It is strange that there is no published analysis as to why OPV production in the Indian public sector has failed repeatedly while Panacea Biotec, an Indian private sector firm, has recently secured a comfortable position as a WHO pre-qualified supplier of OPV for UNICEF. Panacea repackages its OPV from imported bulk OPV obtained from Biopharma (Bandung, Indonesia) and Chiron (Siena, Italy). Some argue that even though India always had an effective indigenous injectable polio vaccine (IPV), OPV was recommended in developing countries because international organizations were trying to find new markets for United States multinational corporations (since market demand for OPV ceased to exist in the US and other developed countries by the end of the 20th century) [7].

The failure of the Indian public sector in vaccine production was not limited to OPV. In 1984, the government took over Bengal Immunity Ltd. (Kolkata, India), a loss-making private company, and revived it so that it could supply TT, DT, DPT and other products to the government. But within a decade, the government declared that the company was financially unviable and eventually closed it. Similarly, in 1989, the Union government's Department of Biotechnology established a new public sector unit, Indian Vaccine Corporation Ltd. (IVCOL) at Gurgaon, for the indigenous production of measles vaccine with technology transferred from Institut Merieux, a public sector company based in Lyons, France. However, the technology transfer never materialised, as the private sector took over the French public sector firm and denied the technology transfer to IVCOL [8]. IVCOL was eventually closed down, and India's entire measles vaccination requirement was met through imports until an Indian private company based in Pune, the Serum Institute of India (SII), started its supply to the EPI in 1992.

The inability of the Indian public sector to recover from its mounting failures to achieve self-sufficiency and self-reliance in primary vaccines is also related to the liberalisation and globalisation of the Indian economy. It is not a coincidence that these failures and closures, and the preference for imports (while paying lip service to self-reliance), happened after the Indian government liberalised its economy in 1991 as prescribed by the International Monetary Fund and the World Bank. It is no longer fashionable to produce vaccines in the public sector in India, let alone to try and revive failing public sector units, even if essential vaccines are not available from the private sector.

## The Increased Role of the Private Sector: Distorted Prioritisation of Vaccine R&D/Production

**Figure 1:**
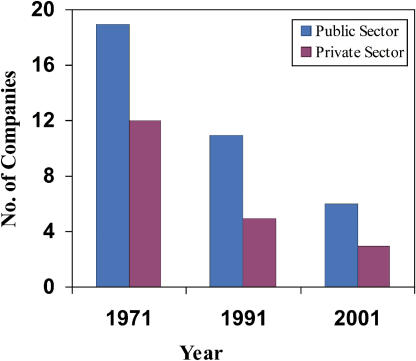
Primary Vaccine Suppliers to the Indian EPI in the Last Four Decades The data were compiled from the annual reports of Health Information of India (1970–1971 to 2001–2002), and the Ministry of Health and Family Welfare, Government of India, New Delhi.

**Figure 2:**
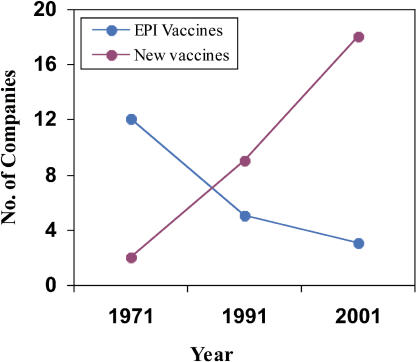
The Growth of the Private Sector in the Indian Vaccine Market The data were compiled from the annual reports of Health Information of India (1970–1971 to 2001–2002), the Ministry of Health and Family Welfare (Government of India) and MIMS India (www.mims-india.com), Nov 2001, New Delhi.

One of the main reasons for the growing gap in demand for and supply of primary vaccines in India is that while public sector production is on the decline (Figure 1), vaccine availability from the private sector (Figures 1 and 2) or through the UNICEF procurement mechanism (based on global tenders from suppliers pre-approved by the WHO) has not improved. This is a part of a worrisome global trend that has been acknowledged by UNICEF (http://www.unicef.org/supply/index_vaccine_security.html).

Shortages of primary vaccines in developing countries began to emerge in the late 1990s. These shortages were due to the introduction of new, more sophisticated, more expensive vaccines in industrialised country markets, leading to manufacturers phasing out the production of the traditional, less expensive vaccines used in developing countries. Between 1998 and 2001, ten out of 14 major manufacturers partially or totally stopped production of traditional vaccines. Eight of these firms were the main suppliers of vaccines to UNICEF. Of these eight, six were involved in mergers between larger pharmaceutical companies. The overall outcome of these developments is that the availability of primary vaccines to UNICEF has dramatically decreased, while the prices have increased (http://www.unicef.org/publications/index_4442.html).

Indeed, the rapid growth (8%–10% per annum) of India's current human vaccine market is mainly attributed to the new, high-priced vaccines (Figure 2 and Table 2) such as Hepatitis B that have been launched since the 1990s. There has been pressure from the industry to include these new vaccines in the government's UIP, even though the clinical and epidemiological justification for their inclusion is controversial [9,10]. With epidemiology taking a backseat, government decisions on vaccination are increasingly determined by price competition and supply “push” (by the companies) rather than “pull” (demand) from proven public health needs [9].

**Table 2:**
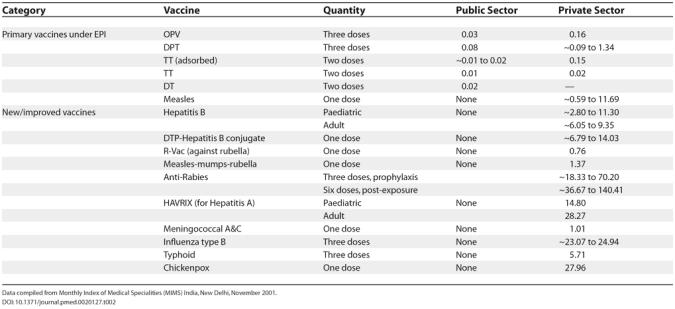
Cost of Full Immunisation with Each Vaccine (in US dollars)

Data compiled from Monthly Index of Medical Specialities (MIMS) India, New Delhi, November 2001.

Many western countries have included several other new vaccines (such as influenza type B, meningitis, measles-mumps-rubella, and chickenpox) in their regular immunisation programmes [11]. These trends are used as a justification by the industry to include these vaccines in the Indian UIP in the future. Aggressive promotional campaigns for the new vaccines and their quick adoption by industry-friendly private medical practitioners have already made these vaccines akin to fast-moving consumer goods. The industry, which enjoys all the benefits of economic liberalisation, sees no contradiction in seeking a captive market for its new vaccines through the government-sponsored UIP while at the same time failing to meet its social responsibility to meet the shortfall in production of existing UIP vaccines.

There is another serious contradiction that grips the global drug and vaccine industry. For curative medicine, the pharmaceutical industry places increasing emphasis on the use of genomics and bioinformatics to move toward customised medicine to suit different populations. And yet in vaccines, the tendency is to move toward a “one vaccine fits all” regime. This would be fine if the vaccines were specifically designed for universal use, but there was no attempt to conclusively establish that the imported vaccines actually suited the Indian strains of the pathogens before they were adopted. Doubts over suitability that have subsequently emerged have not been adequately addressed. With the decline of epidemiology and disease surveillance in India, and the main emphasis being on the statistics of vaccine “coverage” rather than the immune protection achieved, it seems that spending money on vaccines is more important than actual disease prevention. If these trends continue unabated, they will lead to serious distortions in the vaccination programmes of India and other developing countries facing a similar situation.

## Conclusions and Recommendations

India enjoyed the advantages of early initial successes in vaccine R&D and indigenous production in the public sector, but the country is increasingly unable to cope with the growing gap in the demand and supply of UIP vaccines [6]. The availability of UIP vaccines from the private sector is also on the decline in India and abroad, in favour of more expensive new vaccines and combination vaccines, whose public health need has not been unequivocally established in India with sound epidemiological and cost-benefit data [9,12]. Therefore, India (and indeed, every country) must evolve its own national strategies to meet its vaccination needs within its budgetary constraints. To do so will require four key actions.

The first and foremost element in this strategy must be the decisive intervention of the Indian government to meet the shortfall in the UIP vaccines. This may be done either by strengthening the public sector wherever possible, or by taking suitable (and transparent) measures to encourage the indigenous private sector on a case-by-case basis to make safe and effective vaccines available at affordable prices. The suitability of imported vaccines to deal with Indian pathogenic strains also needs to be conclusively established wherever necessary. The health security of a nation of India's size cannot be left to the vagaries of global market forces. With a strong will and a small amount of planning, the current situation in India can be reversed, and India can even play a major role in meeting the global shortfall in the vaccines procured by UNICEF.

Secondly, India needs to strengthen epidemiology and revive the collapsing disease surveillance system. This would help to decide between universal or selective immunisation based on unequivocal scientific evidence, as well as to respond to the changing disease prevalence scenario on the ground, which may call for a move from universal to selective immunisation or vice versa. Some diseases may not need vaccinating against at all, and may be better controlled by other strategies, such as better sanitation, vector control, quarantine, and curative medicines. National immunisation programmes must be led by scientifically established public health needs and not by the mere availability of a vaccine in the market.

Thirdly, a strong emphasis on in-house R&D is needed in order to ensure that our production technologies are in tune with the times, and to negotiate strategic partnerships with outside scientists or institutions and companies.

Last but not least, the Indian government should actively encourage independent policy research, cost-benefit studies, and wider national consultations on various aspects of vaccination and public health so that it can take more informed decisions on such matters.

## Abbreviations

DPT: diphtheria
DT: diphtheria toxoid
EPI: Expanded Programme of Immunization
IPV: injectable polio vaccine
IVCOL: Indian Vaccine Corporation Ltd.
OPV: oral polio vaccine
SII: Serum Institute of India
TT: tetanus toxoid
UIP: Universal Immunization Programme
UNICEF: United Nations Children's Fund
WHO: World Health Organization
